# Characterization of a recombinant Sendai virus vector encoding the small ruminant lentivirus *gag*-P25: antiviral properties in vitro and transgene expression in sheep

**DOI:** 10.1186/s13567-025-01475-2

**Published:** 2025-03-07

**Authors:** Álex Gómez, Idoia Glaria, Irati Moncayola, Irache Echeverría, Javier Arrizabalaga, Ana Rodríguez-Largo, Ignacio de Blas, Delia Lacasta, Estela Pérez, Marta Pérez, Alicia De Diego, Ricardo De-Miguel, Benhur Lee, Lluís Luján, Ramsés Reina

**Affiliations:** 1 Departamento de Patología Animal, Universidad de Zaragoza, Zaragoza, Spain; 2 Instituto Agroalimentario de Aragón-IA2, Universidad de Zaragoza, Zaragoza, Spain; 3 Instituto de Agrobiotecnología (CSIC-Gobierno de Navarra), Mutilva, Navarra Spain; 4 Departamento de Agronomía, Biotecnología y Alimentación, Universidad Pública de Navarra, Pamplona, Spain; 5 Departamento de Anatomía, Embriología y Genética Animal, Universidad de Zaragoza, Zaragoza, Spain; 6 Centro de Investigación Biomédica de Aragón (CIBA), Instituto Aragonés de Ciencias de La Salud (IACS), Zaragoza, Spain; 7 AnaPath Services GmbH, Liestal, Switzerland; 8 Department of Microbiology, Icahn School of Medicine at Mount Sinai, New York, NY USA

**Keywords:** Sendai, viral vector, vaccine, small ruminant lentiviruses, innate immunity

## Abstract

Small ruminant lentiviruses (SRLV) cause multisystemic chronic inflammatory disease and significant economic losses in sheep and goats worldwide. However, no vaccines or therapies are currently available. In this study, a recombinant Sendai virus (SeV) vector encoding the SRLV *gag*-P25 gene (rSeV-GFP-P25) from the EV1 strain was generated using In-FUSION cloning and rescued using the SeV reverse genetic system. Transgene expression and stimulation of innate immunity and interferon-stimulated genes (ovine A3Z1, OBST2 and SAMHD1) were evaluated in ovine skin fibroblasts (OSF) transduced with SeV-GFP and rSeV-GFP-P25. Additionally, to characterize the effect of the SRLV restriction in transduced OSF, the SRLV DNA load was quantified at different times post-transduction and post-infection with strain EV1. Using immunohistochemistry and image analysis, transgene expression and tissue distribution of recombinant P25 were studied in two lambs inoculated intranasally, one with rSeV-GFP-P25 and the other with SeV-GFP. rSeV-GFP-P25 induced efficient and transient transgene expression in vitro and in vivo. Furthermore, OSF transduced with rSeV-GFP-P25 presented upregulation of TLR2, TLR3, TLR6, TLR7, RIG-I, MyD88 and IFN-β, whereas SeV-GFP did not induce TLR6 or IFN-β upregulation. Among the interferon-stimulated genes, OBST2 was significantly upregulated after transduction with rSeV-GFP-P25 compared with the empty vector. SRLV restriction gradually increased and persisted after transduction with SeV-GFP and rSeV-GFP-P25, with OSF transduced three times showing cumulative restriction. Forty-eight hours post-inoculation in vivo, marked P25 expression was observed in ciliated epithelial cells and submucosal macrophages/dendritic cells of the nasal mucosa. This study reinforces the important role of the innate immune response in controlling SRLV infection and suggests that rSeV-GFP-P25 is a potential vaccine candidate against SRLV.

**Supplementary Information:**

The online version contains supplementary material available at 10.1186/s13567-025-01475-2.

## Introduction

Small ruminant lentiviruses (SRLV) are the cause of a globally distributed and highly contagious viral disease that affects mainly sheep and goats, triggering a multisystemic chronic inflammatory condition and limiting animal production and trade [1–4]. SRLV include Maedi-Visna virus (MVV) and caprine arthritis encephalitis virus (CAEV), two viruses with a wide spectrum of strains capable of crossing the sheep/goat barrier [5, 6]. High genetic heterogeneity is a hallmark of SRLV infection, primarily due to the lack of exonuclease activity in the reverse transcriptase of the virus. This jeopardizes diagnostic and vaccination strategies, promoting immune escape variants [7–10].

SRLV target cells are involved in orchestrating innate and adaptive immune responses, such as monocyte‒macrophage lineage and dendritic cells (DCs). The innate immune response plays a crucial role in SRLV infection after recognition by Toll-like receptors (TLR) 7/8, modulating the expression of multiple cytokines [11–14]. TLR 7/8 SRLV-mediated activation can induce the production of a mixture of interferons (IFN) type I (IFN-α and IFN-β) and type II (IFN-γ) through downstream signalling molecules [15, 16]. IFN type I is produced by numerous cell types after virus infection and mediates the expression of IFN-stimulated genes (ISGs) [16, 17]. SRLV-induced IFN-I can inhibit virus replication by upregulating ISGs, producing molecules such as interferon-induced transmembrane protein 3 (IFITM3), SAM domain and HD domain-containing protein 1 (SAMHD1), tripartite motif-containing protein 5 alpha (TRIM5α), catalytic polypeptide-like 3 (APOBEC3) and ovine BST2 (OBST2/Tetherin) [17–20]. Additionally, the innate immune response can modulate the adaptive immune response through IFN-γ production, determining the transcription profile of macrophages or stimulating NK cell activation [20, 21]. However, lentiviruses can inhibit the IFN-mediated response and promote the degradation of antiviral factors induced by IFN [22–24].

Various types of vaccine candidates have been tested against SRLV, including live attenuated, inactivated, subunit, plasmid and Vaccinia virus-based vaccines. However, to date, no vaccines or therapies are available [25]. Live attenuated vaccines with deletions in the *vif* and *tat* genes may confer protection against homologous SRLV but can potentially revert to pathogenicity [26–29]. Inactivated SRLV vaccines have traditionally exacerbated the disease [30, 31], but more recently, a formalin-inactivated whole MVV vaccine has shown partial protection against natural SRLV infection in lambs [32]. Subunit vaccines based on immunodominant proteins, such as the CAEV recombinant gp135 surface glycoprotein, induce neutralizing antibodies; however, the CAEV *gag* proteins favour SRLV replication [33, 34]. Plasmid DNA immunizations encoding the *env* and *gag* genes have shown a strong humoral response and early restriction of SRLV replication, leading to milder disease after challenge [35–38]. These vaccines confer short-term protection and sometimes facilitate SRLV infectivity [36, 37]. Vaccinia-based vectors encoding the *env* gene have shown robust humoral and cellular responses but not complete protection against challenge [39, 40]. Heterologous nasal and systemic prime-boost regimens involving plasmid DNA and vaccinia-based vectors, both encoding the *env* and *gag* genes, have shown protective effects after challenge, reducing the proviral load and lesion severity [40, 41]. The SRLV P25 protein, a major core protein encoded by the *gag* gene, has immunogenic properties, generating high titres of functional antibodies, and has been suggested as a vaccine antigen candidate [42, 43].

Sendai virus (SeV) vectors-based vaccines have shown promising safety and efficacy profiles in the development of vaccine prototypes for primate lentiviruses, such as human immunodeficiency virus (HIV) and simian immunodeficiency virus (SIV) [44–46]. SeV vectors have demonstrated efficient transgene expression in sheep, robust type I IFN-mediated innate immune response activation and partial restriction against SRLV infection in sheep cells in vitro [47, 48]. SeV vectors could be an alternative strategy to develop a new generation of safe and effective vaccines against SRLV infection in small ruminants.

In this study, we generated a recombinant Sendai virus (SeV) vector encoding the SRLV *gag*-P25 gene (rSeV-GFP-p25) and studied transgene expression, the innate immune response and antiviral activity against SRLV infection in ovine skin fibroblasts*.* Additionally, transgene expression and tissue distribution were analysed in two lambs after SeV-GFP and rSeV-GFP-P25 intranasal inoculation.

## Materials and methods

### Cells, plasmids and viruses

For cell cultures, ovine skin fibroblasts (OSF) were obtained from skin biopsies of SRLV-free lambs. These animals were seronegative to two commercially available ELISA kits: the Elitest-MVV (Hyphen-Biomed, France) and Eradikit™ SRLV screening kits (IN3 diagnostic, Italy) [49, 50] and negative to PCRs *Gag*-Pol and Craft-Oslo (Additional file 1). OSF and human embryonic kidney cells (HEK293T) were incubated at 37 °C with 5% CO_2_ in Dulbecco’s modified Eagle’s medium (DMEM) (Deltalab, Spain) supplemented with 10% heat-inactivated fetal bovine serum (FBS), 1% L-glutamine and a 1% antibiotic/antimycotic mixture (Sigma Aldrich, St. Louis, Missouri, USA).

Sendai virus (SeV) antigenome plasmids containing green fluorescent protein (GFP) (SeV-GFP) and accessory plasmids (T7-SeV-N, T7-SeV-P, T7-SeV-L and T7opt) were used [54]. SeV-GFP derives from the SeV Fushimi strain with mutations introduced into the F and M genes, as previously described [51].

SRLV viral stocks from genotype A (strain EV1) [52] were propagated on OSF, and supernatants were collected when approximately 90% of the culture showed syncytia. SRLV was subsequently titrated on 96-well OSF culture plates using the Reed–Muench method [53]. The titre was calculated as the 50% tissue culture infectious dose per millilitre (TCID_50_/mL) and was used for in vitro infections at the specified multiplicity of infection (MOI).

### Construction and viral rescue of recombinant SeV

The SRLV *gag*-P25 gene sequence was amplified from the SRLV strain EV1 and cloned and inserted into the SeV-GFP plasmid by In-FUSION cloning between the Gaussia-Dura Luc and GFP genes (primers in Additional file 1; In-Fusion HD Cloning Kit; Takara Bio, USA, Inc.), generating the recombinant plasmid rSeV-GFP-P25. Correct cloning was checked by sequencing.

The SeV reverse genetics system was used for SeV-GFP and rSeV-GFP-P25 rescue [54]. Briefly, 0.5 µg of antigenomic SeV-GFP or rSeV-GFP-P25 and accessory plasmids (0.173 µg of T7-SeV-N, 0.1 µg of T7-SeV-P, 0.01 µg of T7-SeV-L, 0.5 µg of T7opt) were co-transfected into 60–70% confluent HEK293T cells using jetPRIME^®^ transfection reagent (Polyplus) following the manufacturer’s instructions (1:2 ratio). The cell transfection efficiency was monitored by fluorescence microscopy (Nikon Eclipse TE300) to detect virus-encoded GFP fluorescence. GFP-positive cell culture supernatants were collected at 96 h post-transfection, clarified by centrifugation at 2500 rpm for 5 min and stored at −80 °C. For viral production, these supernatants were propagated at different MOI by successive passages in HEK293T cell cultures. Finally, SeV-GFP and rSeV-GFP-P25 viral stocks were titrated into HEK293T cells by fluorescence examination in 96-well culture plates using the Reed-Muench method [53].

### Relative mRNA expression quantification in OSF

OSF were transduced with SeV-GFP or rSeV-GFP-P25 at 1 MOI, and cell lysates were collected at 12, 24, 48, 72, 96 and 120 h post-transduction (hpt). mRNA extraction was performed automatically (NucleoMag RNA-Magnetapure 32, Dominique Dutscher), followed by retrotranscription to cDNA using random hexamers and oligo (dT) primers (PrimeScript RT Reagent Kit, Takara Bio, Kyoto, Japan). P25 transgene expression was evaluated at 24, 48, 72, 96 and 120 hpt. Additionally, to evaluate the innate immune response stimulated in vitro by SeV-GFP and rSeV-GFP-P25, the expression of different Toll-like receptors (TLR1-10), retinoic acid-inducible gene I (RIG-I), the adaptor MyD88 and interferon β (IFN-β) was measured at 12, 24, 48 and 72 hpt. cDNA amplification was performed by quantitative polymerase chain reaction (qPCR) on an AriaMx Real-time PCR System (Agilent Technologies, Santa Clara, CA, USA) using SYBR Premix Ex Taq (Takara, Kyoto, Japan) with P25-specific primers (Additional file 1). β-actin was used as a housekeeping gene for relative quantification (2^−ΔCt^ method). To evaluate TLRs, RIG-I, MyD88 and IFN-β upregulation, basal gene expression was measured in OSF under standard in vitro conditions without any transduction or infection and used as a threshold value for relative quantification (fold change, 2^−ΔΔCt^ method). Genes with values higher than 1 were considered upregulated on a logarithmic scale.

OSF were transduced with SeV-GFP or rSeV-GFP-P25 at 1 MOI, and cell lysates were collected at 24, 48 and 72 hpt. Ovine A3Z1, ovine BST2 (OBST2) and SAMHD1 antiviral factor expression was evaluated, as described previously, using specific primers (Additional file 1). A logarithmic scale and the 2^−ΔΔCt^ method were used, as described above.

### SRLV restriction in OSF

The ability of the two SeV-based vectors to restrict infection by the SRLV strain EV1 was evaluated in vitro. First, we analysed the viral restriction capacity as a function of the time post transduction. OSF plated at 2 × 10^5^ per well in 12-well plates were transduced with SeV-GFP or rSeV-GFP-P25 at 1 MOI. Twenty-four, 48, 72 and 96 hpt OSF were infected with the EV1 strain at 0.5 MOI. Twenty-four hours post-infection (hpi) with the strain EV1, cell lysates were collected, and nucleic acid extraction was performed manually (E.Z.N.A.® Blood DNA Kit, Omega Biotek). To evaluate viral restriction induced by SeV-GFP and rSeV-GFP-P25, the viral DNA load was measured by qPCR on an AriaMx real-time PCR system (Agilent Technologies, Santa Clara, CA, USA) with specific primers and probes for strain EV1 (Additional file 1).

Second, the persistence of viral restriction induced by viral vectors after infection with EV1 was studied. OSF plated at 2 × 10^5^ cells per well in 12-well plates were transduced with SeV-GFP or rSeV-GFP-P25 at 1 MOI. At ninety-six hpt, OSF were infected with EV1 at 0.5 MOI. Twenty-four, 48 and 72 hpi, the cell lysates were collected, and the EV1 viral load was measured as described above.

Third, SRLV infection restriction after three consecutive transductions by SeV-GFP or rSeV-GFP-P25 was studied. OSF plated at 2 × 10^5^ cells per well in 12-well plates were transduced with SeV-GFP or rSeV-GFP-P25 at 1 MOI once, twice or thrice, with a period of 24 h between transductions. Twenty-four hours after the last transduction, OSF were infected with the strain EV1 at 0.5 MOI. Twenty-four hpi cell lysates were collected, and the EV1 viral load was measured as described above. In the three experiments, untreated OSF infected with EV1 at the mentioned times were used as positive controls, and a standard curve (R^2^: 0.98) was used to determine the number of SRLV copies per nanogram of DNA.

### Antiviral activity bioassay

The antiviral activity of supernatants collected at 24, 48 and 72 hpt after OSF transduction with SeV-GFP or rSeV-GFP-P25 at 1 MOI was evaluated by incubation with fresh OSF cells. After 24 h of incubation with these supernatants, OSF were infected with strain EV1 at 0.5 MOI. Twenty-four hpi, the cells were collected, and the EV1 viral load was measured as described above. The positive control consisted of OSF infected with EV1. A standard curve (R^2^: 0.98) was used to determine the number of SRLV copies per nanogram of DNA.

### Immunocytochemical evaluation of transgene expression in OSF

OSF plated at 10^6^ cells per well in 6-well plates were incubated for 24 h at 37 °C with 5% CO_2_ in DMEM and transduced with SeV-GFP or rSeV-GFP-P25 at 1 MOI. Twenty-four, 48, 72 and 96 hpt, OSF were washed with PBS and fixed with a mixture of methanol and 5% hydrogen peroxide for 10 min at room temperature (RT). Following 3 × PBS washes, OSF were incubated with the undiluted monoclonal antibody VPM70 against the capsid viral protein P25 for 90 min at 4 °C [42]. After 4 washes, the cells were labelled with an HRP-conjugated sheep anti-mouse antibody (1:2000) for 45 min at 4 °C. Immunostaining was visualized with 3–3’-diaminobenzidine (DAB)-H_2_O_2_ under optical microscopy. Untreated OSF under in vitro conditions were used as a negative control, and untreated OSF infected with EV1 were used as a positive control.

### Animals and experimental immunization

The animal experiments were performed at the Veterinary Faculty of Zaragoza. The Ethical Committee of the University of Zaragoza approved and licenced all the experimental procedures (ref: PI43/18). The requirements of the Spanish Policy for Animal Protection (RED53/2013) and the European Union Directive 2010/63 on the protection of experimental animals were always met.

Two 8-month-old *Rasa Aragonesa* breed lambs were selected from the ruminant clinic service (SCRUM, Veterinary Faculty of the University of Zaragoza). They were housed in separate boxes under the same conditions. The lambs tested seronegative for the Eradikit™ SRLV screening kit (IN3 diagnostic, Italy) [50] and negative for PCR with the primers *Gag*-*Pol* and Craft-Oslo (Additional file 1) [55, 56]. A 2 mL dose of 10^7^ TCID_50_ of either rSeV-GFP-P25 or SeV-GFP was used for experimental inoculation of each lamb. Both lambs were intranasally inoculated using a nebulizer. Forty-eight hours after inoculation, the animals were humanely euthanized and necropsied. The lamb inoculated with SeV-GFP was used as a negative control.

### Histopathological evaluation

During necropsy, SeV target tissues, including nasal mucosa, nasal-associated lymphoid tissue (NALT), trachea, lung (bronchi, bronchiole and alveoli) and mediastinal lymph nodes, were collected [47]. These tissues were fixed in 10% neutral-buffered formalin and embedded in paraffin blocks. Four-micron-thick sections were stained with hematoxylin and eosin (HE) for microscopic evaluation.

### In vivo transgene expression

Samples from the same locations used for histopathological studies were stored at −80 °C until processing. Bronchoalveolar lavage (BAL) fluid was also collected at necropsy as previously described [57]. For nucleic acid extraction, approximately 20 mg of each sample was homogenized with steel beads in a vibrating grinding mill (Mikro-Dismembrator U, Sartorius AG, Germany) coupled with a magnetic bead-based method for RNA extraction and purification (NucleoMag® RNA-MagnetaPure32, MACHEREY–NAGEL, Germany). mRNA extraction and retrotranscription to cDNA were performed as described above. P25 transgene expression was evaluated by qPCR using specific primers (Additional file 1). β-actin was used as a housekeeping gene for relative quantification (2^−ΔCt^ method).

To confirm that the in vivo transgene was expressed as a protein, immunohistochemistry (IHC) for P25 (strain EV1) was performed in all the tissues. Endogenous peroxidase activity was blocked with a 1% hydrogen peroxide solution for 10 min before the sections were incubated for 20 min with EnVision/HRP (EnVision FLEX Mini Kit: K8023, DAKO Agilent). The sections were then incubated for 2 h with a mouse monoclonal anti-P25 antibody (strain EV1, supplied by G.D. Harkiss, University of Edinburgh, United Kingdom) diluted 1:200 (S302281-2, DAKO Agilent). The sections were subsequently incubated with EnVision FLEX + Mouse (K400111-2, DAKO Agilent) for 15 min. Bound antibodies were detected by incubation with DAB (EnVision FLEX Mini Kit: K8023, DAKO Agilent) for 10 min. The sections were then counterstained with hematoxylin (K800821-2, DAKO Agilent) for 8 min. All the incubations were performed at RT. Sections of positive and negative SRLV lungs and mediastinal lymph nodes were used as positive and negative controls, respectively. Additionally, as a negative control for the IHC technique, positive control tissues were incubated with only the antibody diluent.

The IHC anti-P25 slides were Whole Slide Imaged (WSI) scanned by an Axio Z1 (Zeiss, Germany) slide scanner using a Colibri 7 camera and a 20 × objective. Histomorphometry was conducted throughout 2.02 cm of nasal mucosa and submucosa using QuPath version 0.4.4, an open-source software for digital pathology and WSI image analysis [58]. To quantify the amount of transgene expression in the nasal epithelium, the surface of the nasal mucosa with positive immunolabelling against P25 was calculated as follows: (1) Manual annotation of the respiratory cilia with a standardized thickness of 4 μm from the apical surface of epithelial cells; and (2) Application of a machine-learning pixel classifier of random trees to detect positive and negative immunolabelled areas within annotated areas. To quantify transgene uptake by inflammatory cells, positive immunolabelled cells against P25 in the submucosa were calculated using the following methods: (1) manual annotation of the lamina propria and submucosa (excluding tubuloalveolar secretory glands); (2) automated cell detection within annotated areas; and (3) application of a machine-learning object classifier of random trees to detect positive and negative cells.

### Statistical analysis

The data were analysed with IBM SPSS 26.0 for Windows^®^. Each experiment was repeated a minimum of three times. Therefore, continuous variables, such as relative mRNA expression and the number of copies of viral DNA, are presented as the mean and standard deviation. After the Shapiro–Wilk test was used to assess the normal distribution of the data, Levene's test was applied to assess the homogeneity of variance of normally distributed variables. For variables with a normal distribution and equal variances, one-way analysis of variance (ANOVA) or Student’s *t* test was applied. After ANOVA, Bonferroni correction was used for multiple pairwise comparisons. For normally distributed variables and unequal variances, Welch’s t test was applied, and multiple pairwise comparisons were performed via the Games-Howell post hoc test. For non-normally distributed variables, the Kruskal‒Wallis test or Mann‒Whitney U test was conducted. After the Kruskal‒Wallis test, Dunn’s post hoc test was applied for multiple pairwise comparisons. Statistical significance was set at *p* < 0.05.

## Results

### Generation and titration of SeV-based vectors

**Figure 1:**
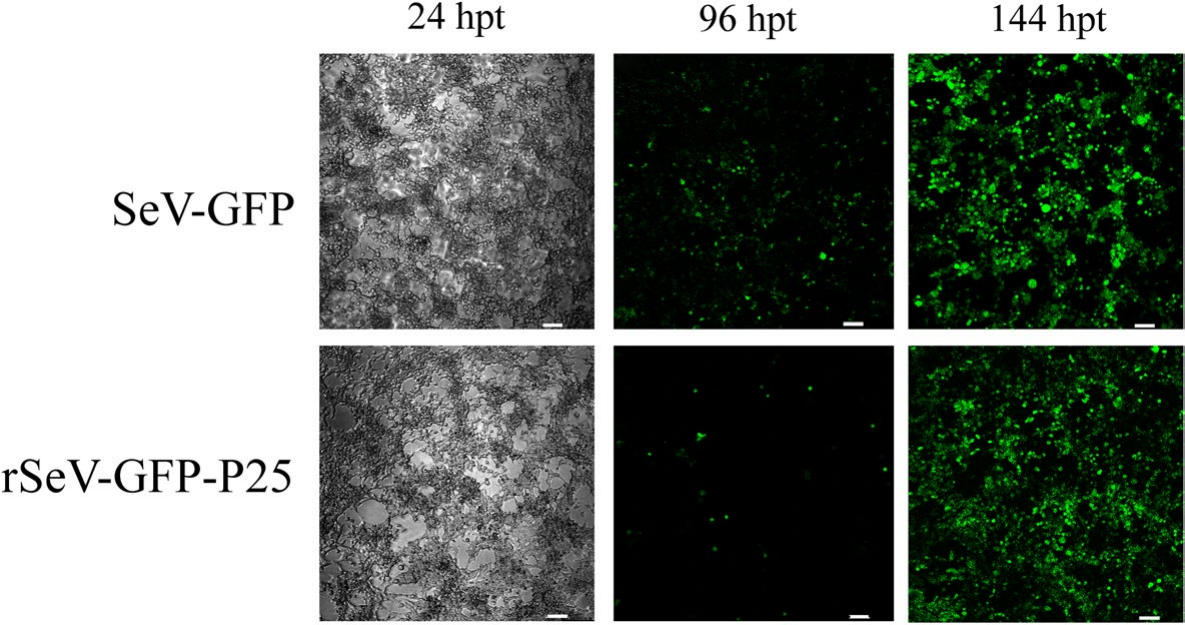
**Transfection of HEK293T cells with SeV-GFP or rSeV-GFP-P25.** The transfection efficiency was determined by calculating the percentage of GFP-positive cells at 24, 96 and 144 h post-transfection (hpt). (Bar = 15 µm).

The transfection of HEK293T cells with SeV-GFP or rSeV-GFP-P25 was confirmed by the presence of GFP-positive cells after fluorescence microscopy evaluation. SeV-GFP transfection resulted in approximately 40–50% GFP-positive cells at 96 h post-transfection, whereas rSeV-GFP-P25 showed a 5–10% transfection efficiency. Both viruses reached approximately 90–100% of GFP-positive cells at 144 h post-transfection (Figure 1). The SeV-GFP and rSeV-GFP-P25 viruses were subsequently collected, filtered and titrated into HEK293T cells using the Reed-Muench method, resulting in 10^6^–10^7^ TCID_50_/mL.

### Stimulation of the innate immune response in OSF

**Figure 2:**
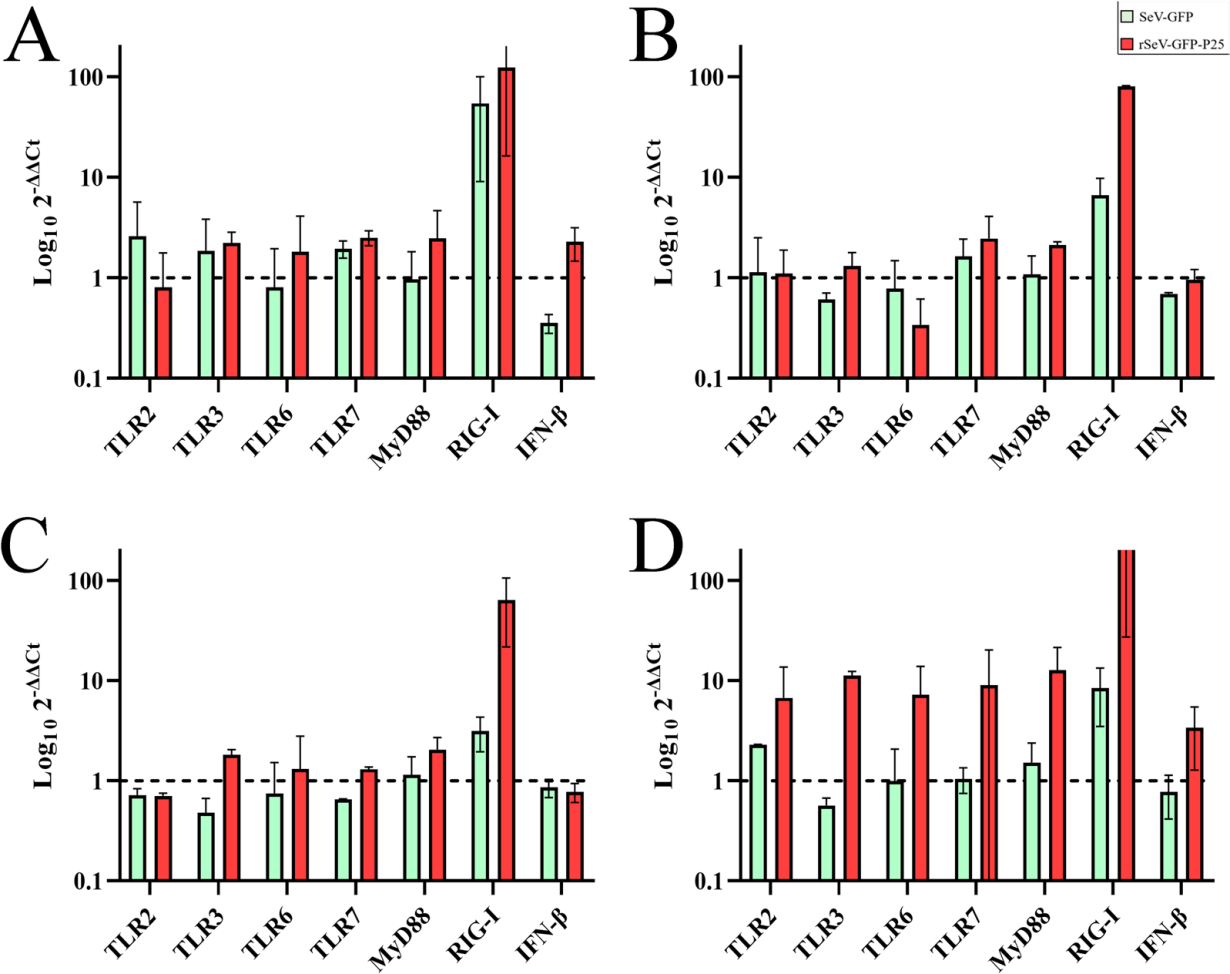
**Quantification of relative mRNA expression of the TLRs, RIG-I, MyD88 and IFN-β genes.** OSF transduced with SeV-GFP or rSeV-GFP-P25 at 12 (**A**), 24 (**B**), 48 (**C**) and 72 (**D**) hours post-transduction. The data shown are the mean and standard deviation.

The mRNA expression of different TLRs, RIG-I, MyD88 and IFN-β genes varied according to the SeV-based vector used and the time after transduction. Compared with OSF transduced with rSeV-GFP-P25, those transduced with SeV-GFP-P25 presented greater upregulation of specific TLRs at all hpt, with the exception of TLR2 at 12, 24 and 48 hpt; TLR6 at 24 hpt; and IFN-β at 48 hpt (Figure 2). Specifically, OSF transduced with rSeV-GFP-P25 overexpressed TLR2, TLR3, TLR6, TLR7, MyD88, RIG-I and IFN-β, whereas SeV-GFP did not induce the upregulation of TLR6 and IFN-β. IFN-β was only upregulated in OSF infected with rSeV-GFP-P25 at 12 and 72 hpt. However, statistically significant differences were not observed between OSF transduced with SeV-GFP and those transduced with rSeV-GFP-P25. RIG-I was the most highly overexpressed gene, peaking in OSF transduced with SeV-GFP at 12 hpt and with rSeV-GFP-P25 at 72 hpt. TLRs 1, 4, 5, 8, 9 and 10 were not differentially expressed compared with the basal expression found in untreated fibroblasts.

### Interferon-stimulated gene expression in OSF

**Figure 3:**
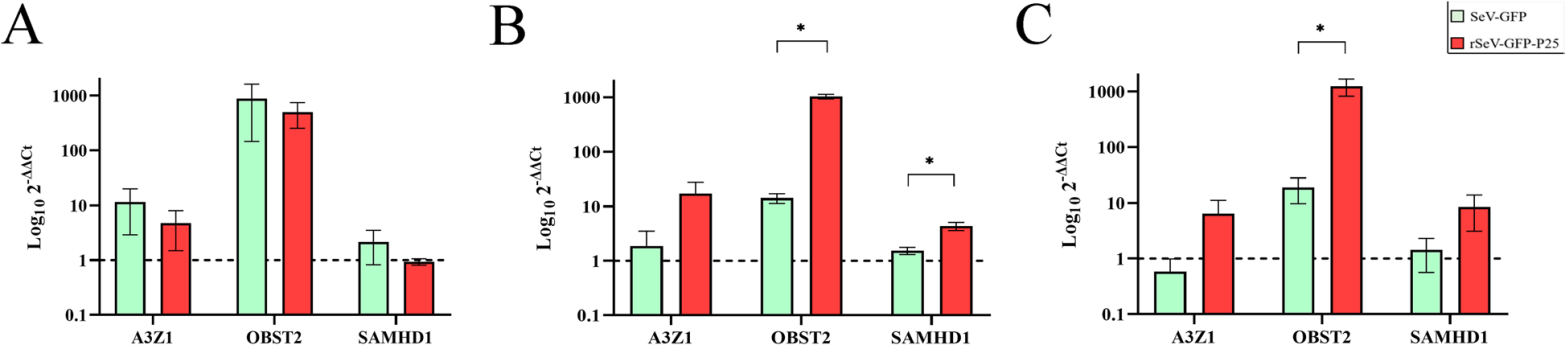
**mRNA relative expression quantification of interferon-stimulated genes (A3Z1, OBST2 and SAMHD1).** OSF transduced with SeV-GFP or rSeV-GFP-P25 at 24 (**A**), 48 (**B**) and 72 (**C**) hours post-transduction (hpt). The data shown are the mean and standard deviation. Statistically significant differences between the groups (*p* < 0.05).

OSF transduced with SeV-GFP and rSeV-GFP-P25 generally overexpressed ovine ISGs (A3Z1, OBST2 and SAMHD1) (Figure 3). Compared with OSF transduced with SeV-GFP, OSF transduced with rSeV-GFP-P25 induced greater upregulation of OBST2 at 48 (*p* < 0.001) and 72 hpt (*p* = 0.002). Additionally, rSeV-GFP-P25 induced greater upregulation of SAMHD1 than SeV-GFP did at 48 hpt (*p* = 0.028). OBST2 was more upregulated than A3Z1 and SAMHD1 in OSF transduced with rSeV-GFP-P25 at 48 (*p* = 0.004; *p* = 0.003) and 72 hpt (*p* = 0.036; *p* = 0.036). Moreover, OBST2 was more highly expressed than A3Z1 and SAMHD1 in OSF transduced with SeV-GFP at 48 hpt (*p* = 0.041; *p* = 0.017).

### SRLV restriction in OSF

**Figure 4:**
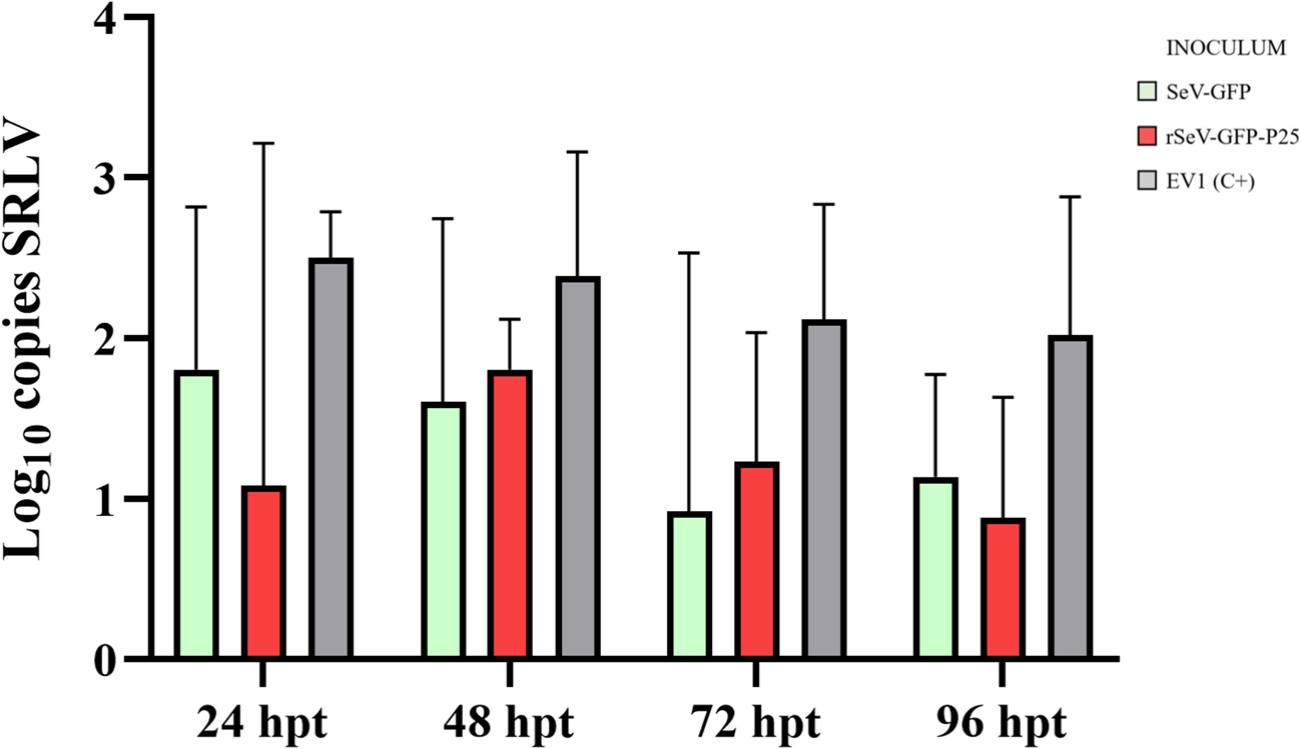
**SRLV DNA quantification (log copies/ng).** OSF were transduced with SeV-GFP or rSeV-GFP-P25 and infected with the SRLV strain EV1 at 24, 48, 72, and 96 h post- transduction (hpt). OSF infected with EV1 at the mentioned times were used as positive controls (EV1 (C+)). The data shown are the mean and standard deviation.

First, the SRLV DNA load was evaluated in OSF previously stimulated with the two SeV-based vectors at different times post transduction (Figure 4). Compared with the positive control, OSF transduced with SeV-GFP or rSeV-GFP-P25 tended to reduce the number of copies of SRLV DNA at all hpt. However, no statistically significant differences were observed.

**Figure 5:**
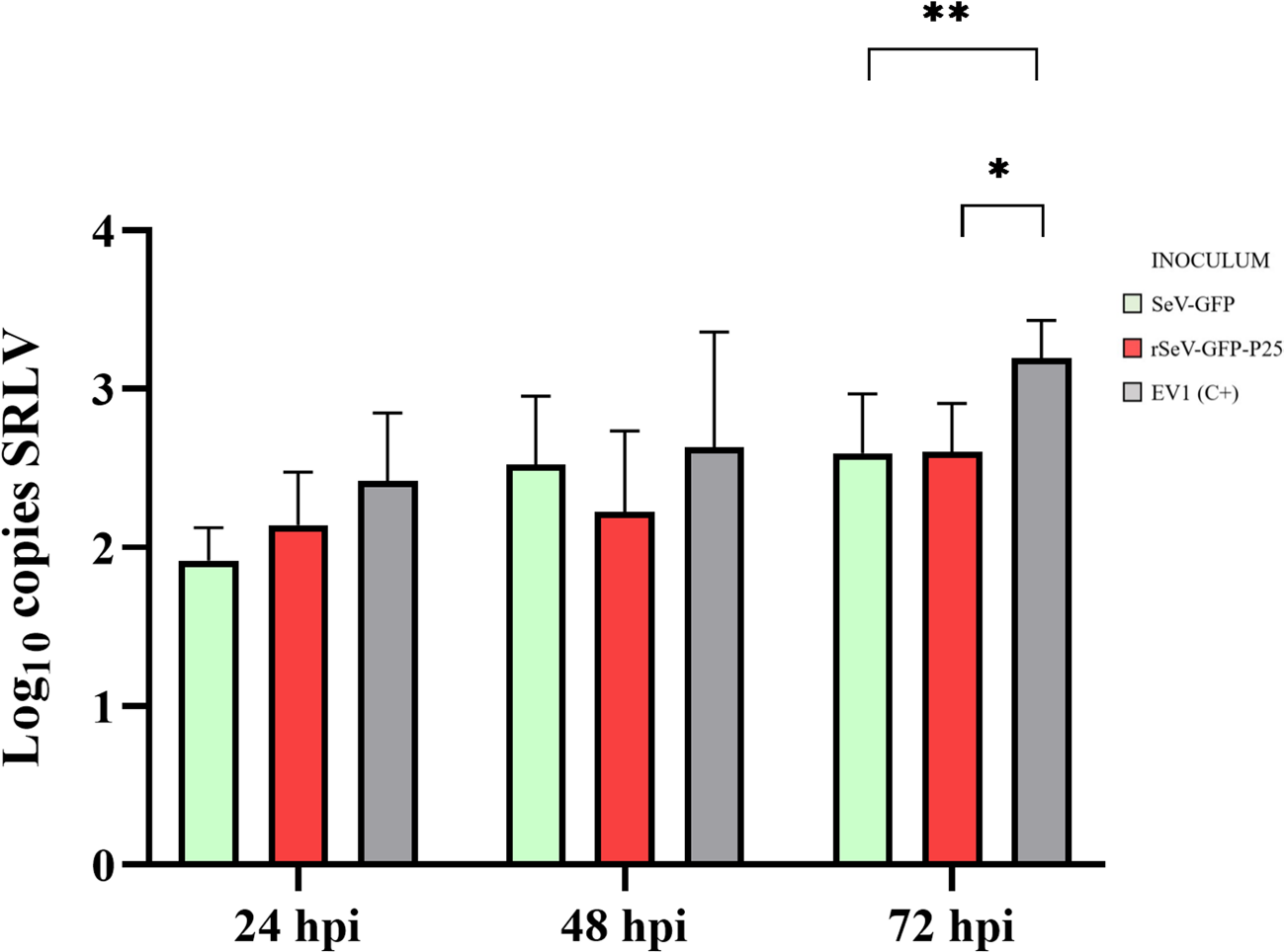
**SRLV DNA quantification (log copies/ng).** OSF infected with the SRLV strain EV1 96 h post-transduction with SeV-GFP or rSeV-GFP-P25 and quantification measured at 24, 48 and 72 h post-infection (hpi). OSF infected with EV1 at the mentioned times were used as positive controls (EV1 (C+)). The data shown are the mean and standard deviation. Statistically significant differences between the groups (*p* = 0.016, **p = 0.015).

Second, OSF were transduced with SeV-GFP or rSeV-GFP-P25 and infected with EV1 at 96 hpt. The SRLV load was then analysed at 24, 48 and 72 hpi (Figure 5). Statistically significant differences were evident at 72 hpi when OSF transduced with SeV-GFP (*p* = 0.015) and rSeV-GFP-P25 (*p* = 0.016) presented lower SRLV viral loads than did the positive control. No statistically significant differences were observed among the inocula.

**Figure 6:**
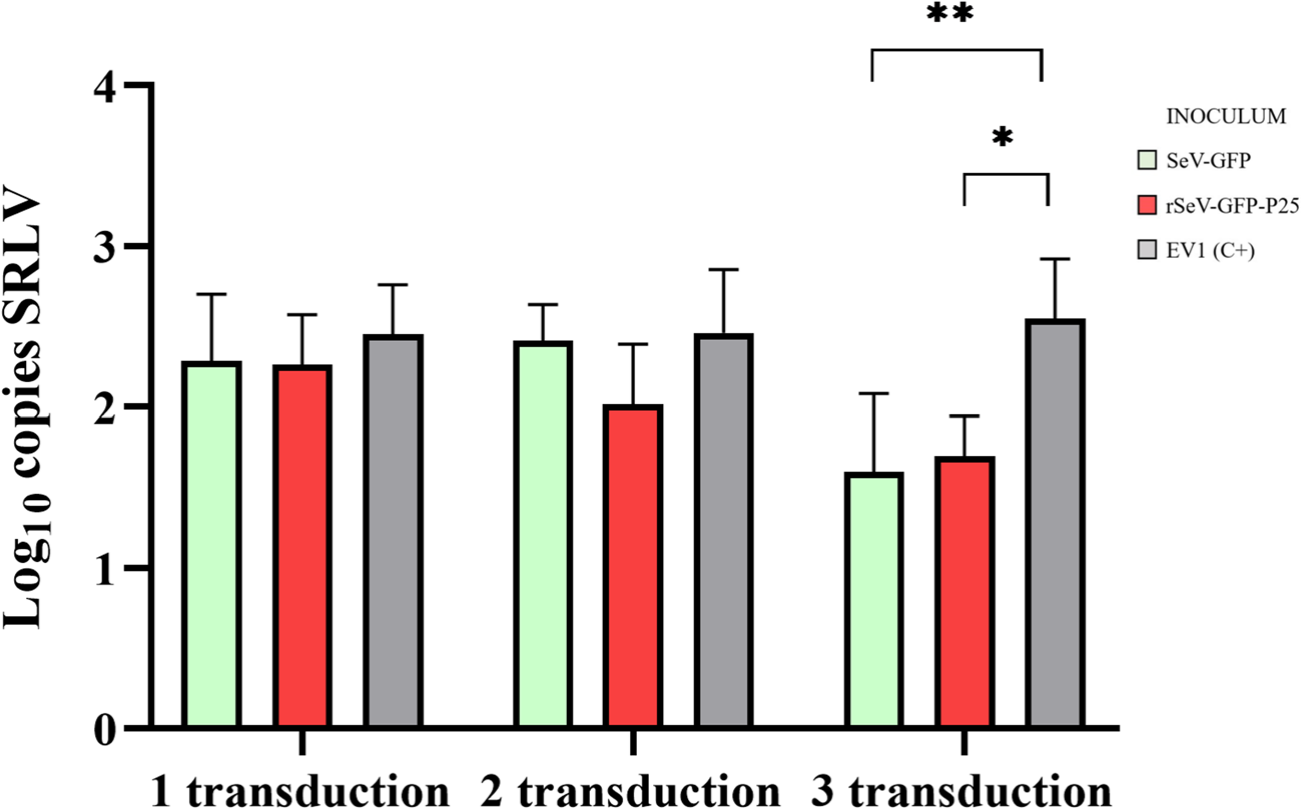
**SRLV DNA quantification (log copies/ng).** OSF were transduced with SeV-GFP or rSeV-GFP-P25 one, two or three times and infected with the SRLV strain EV1 24 h post-transduction. Untransduced OSF and infected with strain EV1 were used as positive controls (EV1 (C+)). The data shown are the mean and standard deviation. There were statistically significant differences between the groups (*p* < 0.05).

Third, the SRLV load was evaluated on the basis of the number of SeV-GFP or rSeV-GFP-P25 transductions (Figure 6). OSF transduced three times presented significantly fewer SRLV DNA copies than did the untransduced control, with significant results for both SeV-GFP (*p* = 0.003) and rSeV-GFP-P25 (*p* = 0.006). Moreover, OSF transduced three times with SeV-GFP presented a significantly lower SRLV load than those transduced with two (*p* = 0.008) or one (*p* = 0.023) transduction agent. On the other hand, OSF transduced three times with rSeV-GFP-P25 presented significantly fewer SRLV DNA copies than those transduced once (*p* = 0.022). No statistically significant differences were detected among the inocula.

### Antiviral activity in OSF

**Figure 7:**
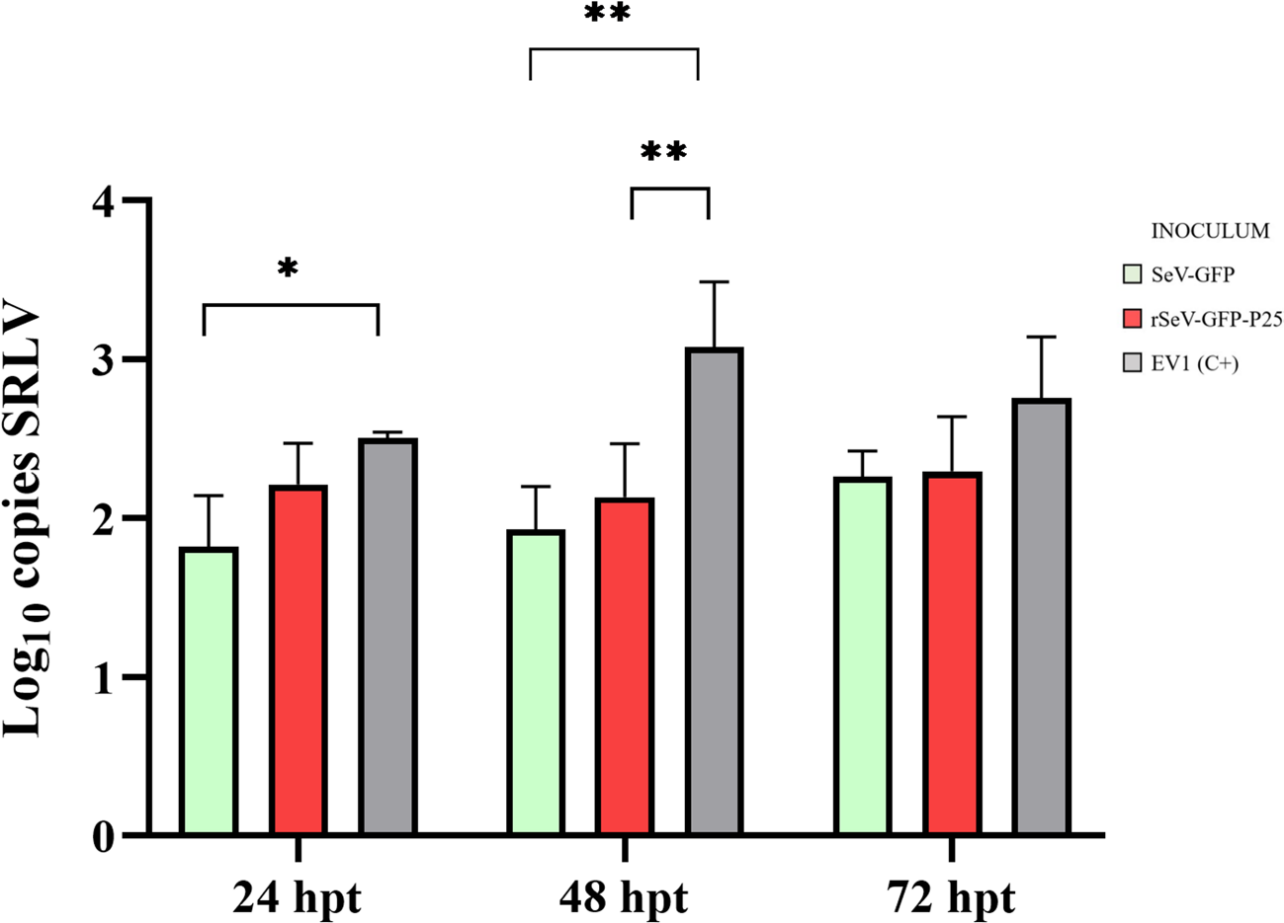
**SRLV DNA quantification (log copies/ng).** OSF were infected with the SRLV strain EV1 and cultured for 24 h with supernatants from OSF transduced with SeV-GFP or rSeV-GFP-P25. The supernatants were collected 24, 48 and 72 h post-transduction (hpt). Untreated OSF infected with EV1 at the mentioned times were used as positive controls (EV1 (C+)). The data shown are the mean and standard deviation. Statistically significant differences between the groups (*p = 0.026; **p < 0.05).

Specifically, supernatant collected 24 hpt from OSF transduced with SeV-GFP (*p* = 0.015) induced significantly lower SRLV DNA copies than did the positive control (Figure 7). Similarly, supernatants collected 48 hpt from OSF transduced with SeV-GFP (*p* = 0.001) or rSeV-GFP-P25 (*p* = 0.005) presented significantly lower SRLV load than did the positive control. These differences persisted at 72 hpt, but no statistically significant differences were detected.

### Transgene expression in OSF

**Figure 8:**
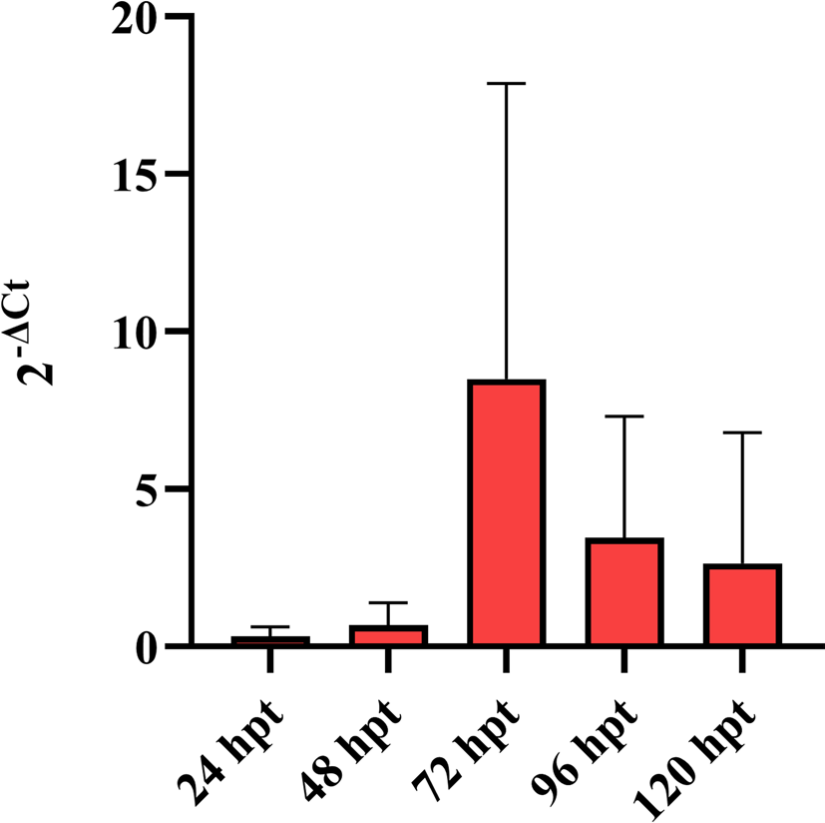
**mRNA relative transgene (*Ggag*-P25) expression quantification in OSF transduced with rSeV-GFP-P25.** The data shown are the mean and standard deviation.

The transduction of OSF with SeV-GFP and rSeV-GFP-P25 was confirmed by the GFP-positive results. Moreover, OSF transduced with rSeV-GFP-P25 showed efficient and progressive transgene expression, peaking at 72 hpt (Figure 8). In the immunocytochemistry assay, positive granular and intracytoplasmic anti-P25 immunolabelling was detected in OSF transduced with rSeV-GFP-P25 at 72 hpt (data not shown).

### Histopathological evaluation

**Figure 9:**
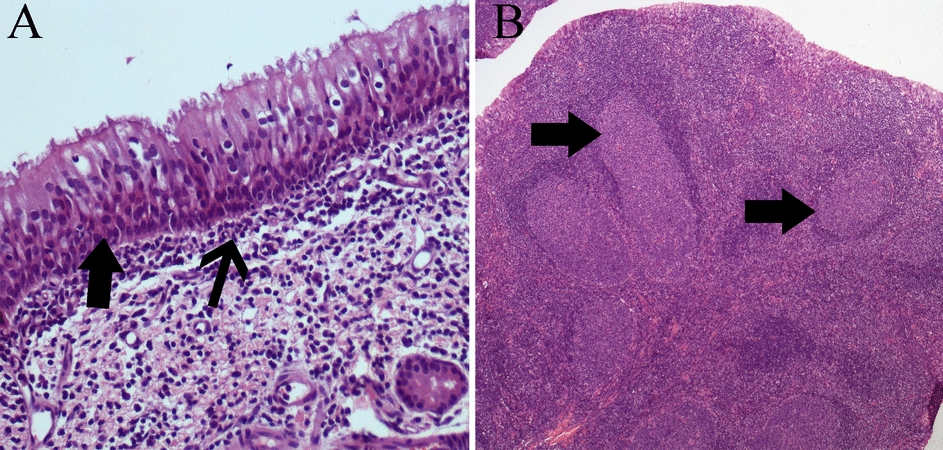
**In vivo histopathological findings after inoculation with rSeV-GFP-P25.** **A** Nasal mucosa: hyperplasia of ciliated epithelial cells (arrow) and submucosal lymphocytic inflammation (thin arrow). Haematoxylin‒eosin (HE). **B** Nasal associated-lymphoid tissue (NALT): reactive lymphoid hyperplasia, characterized by a greater number and size of lymphoid follicles (arrows). HE.

The local host reaction induced by SeV-GFP and rSeV-GFP-P25 intranasal inoculation in lambs was characterized by highly reactive nasal mucosa and NALT (Figure 9). Both inocula induced marked hyperplasia of ciliated epithelial cells and goblet cells of the nasal mucosa, reaching 3–5 layers of cells. Occasionally, single-cell necrosis of epithelial cells was observed. In some areas, the mucosa was infiltrated by a low number of lymphocytes. The submucosa was moderately infiltrated by lymphocytes and fewer neutrophils, macrophages and plasma cells. The submucosa also presented mild neovascularization. NALT showed lymphoid hyperplasia characterized by increased size and number of lymphoid follicles. Changes in the trachea, lung and mediastinal lymph nodes were unremarkable.

### In vivo transgene expression

**Figure 10:**
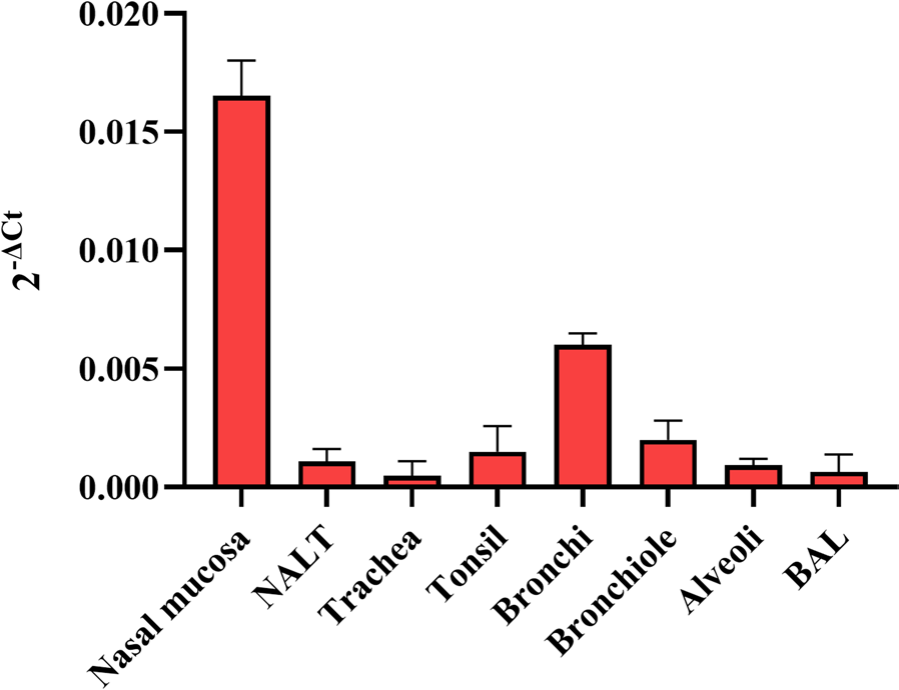
**mRNA relative transgene (*Ggag-P25) expression quantification.*** Tissues were sampled 48 h post-inoculation from a lamb inoculated with rSeV-GFP-P25 (10^7^ TCID_50_). The data shown are the mean and standard deviation. NALT: nasal associated lymphoid tissue, BAL: bronchoalveolar lavage.

Lamb inoculated with rSeV-GFP-P25 presented mild transgene expression in the sampled tissues (Figure 10). The nasal mucosa presented the highest P25 expression, whereas the trachea and BAL fluid presented a lack of transgene expression. Transgene expression in the animals inoculated with SeV-GFP was absent.

**Figure 11:**
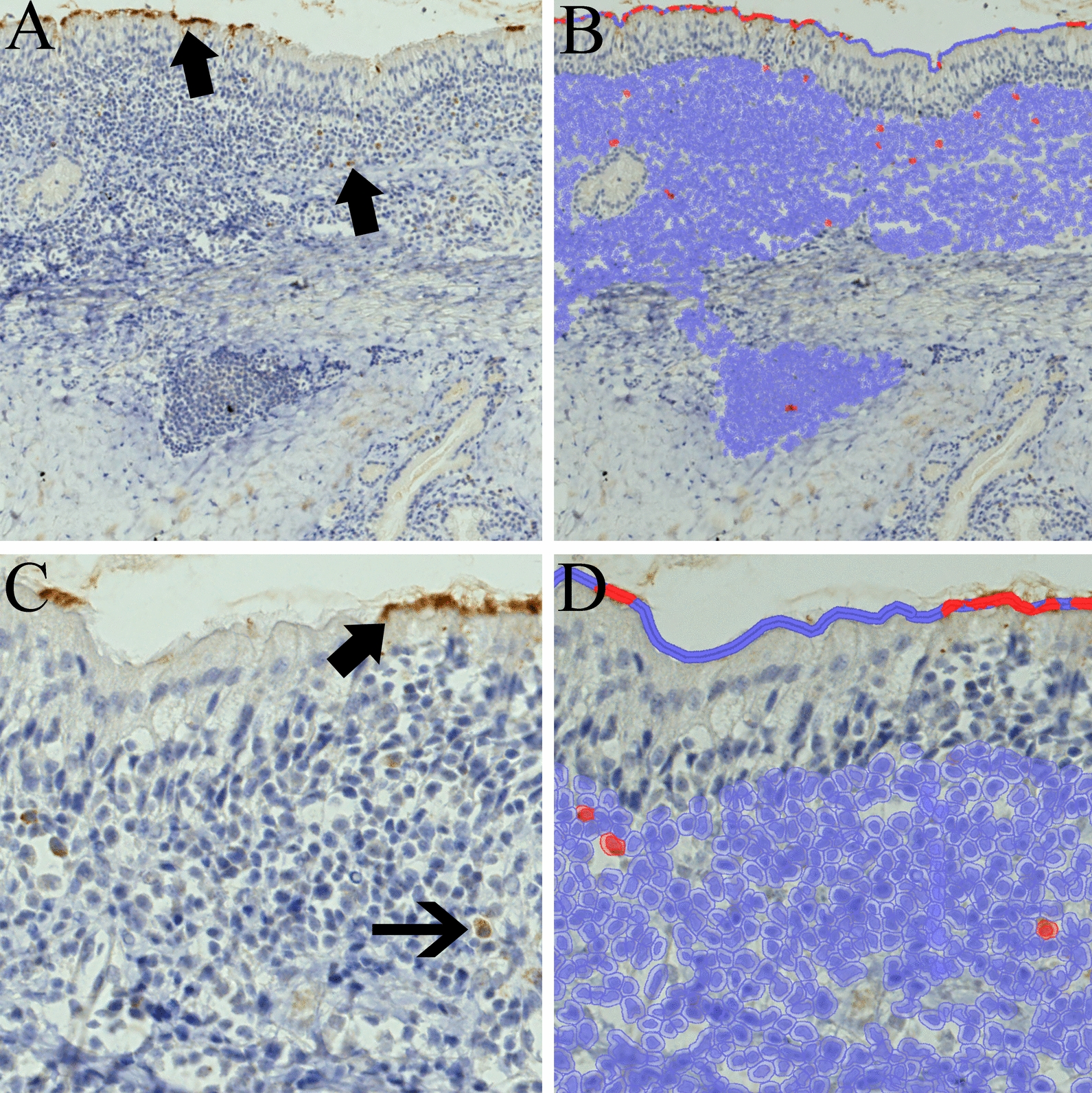
**Immunohistochemistry against the SRLV *gag*-P25 protein (mouse monoclonal anti-P25 antibody).** Nasal mucosa was sampled 48 h post-inoculation from a lamb inoculated with rSeV-GFP-P25. **A** Marked immunoreactivity of ciliated epithelial cells and inflammatory cells in the submucosa (arrows). **B** Image analysis results are shown in blue for the studied areas and in red for the immunolabelled cells. **C** Strong labelling in the apical part of the cilia and cytoplasm of the macrophages/dendritic cells of the submucosa (thin arrow). **D** Image analysis results are shown in blue for the studied areas and in red for the immunolabelled cells.

In lambs inoculated with rSeV-GFP-P25, IHC revealed intense P25 immunoreactivity in the cilia of nasal ciliated epithelial cells (Figure 11). Less frequently, labelling was also present in the inflammatory cells infiltrating the lamina propria and submucosa. Histomorphometry revealed positive immunolabelling of the apical membrane of respiratory cilia in 21.82% of the apical surface of the nasal mucosa from the lamb inoculated with rSeV-GFP-P25. Analysis of the inflammatory cells infiltrating the lamina propria and submucosa revealed 0.19% P25-positive cells. These positive cells presented abundant granular to foamy cytoplasm and intended nuclei and were morphologically compatible with macrophages or DCs. No P25 immunolabelling was observed in the remaining tissues. The lambs inoculated with SeV-GFP were negative for IHC in all the tissues.

## Discussion

Immunization against lentiviruses should involve stimulation of both innate and adaptive responses to effectively control cellular infection. The innate restriction of lentivirus infection has been well documented and ultimately influences the adaptive response, both of which are crucial in limiting SRLV infectivity [12].

In this study, a recombinant SeV vector encoding the SRLV *gag*-P25 gene (rSeV-GFP-P25) was generated using the SeV reverse genetics system [54]. The transduction of OSF was efficient, as evidenced by the GFP-positive results and transgene expression (P25) confirmed by qPCR and immunocytochemistry. Additionally, intranasal inoculation of rSeV-GFP-P25 induced efficient transgene expression in the nasal mucosa and submucosa of one lamb.

rSeV-GFP-P25 induced a strong innate immune response in OSF, partially restricting infection with the SRLV strain EV1. The upregulation of pattern recognition receptors (PRRs) varied according to the SeV-based vector used and the hpt. RIG-I was the most upregulated gene in OSF transduced with SeV-GFP or rSeV-GFP-P25; therefore, RIG-I is likely the predominant factor responsible for the activation of the IFN-mediated response after SeV infection in ovine cells, as described previously in other species [59]. OSF transduced with rSeV-GFP-P25 overexpressed a panel of receptors involved in type-I IFN induction, such as TLR2, TLR3, TLR6, TLR7, MyD88 and RIG-I. Furthermore, IFN-β expression was also confirmed. In contrast, SeV-GFP did not induce the upregulation of TLR6 or IFN-β. TLR2/TLR6 heterodimers sense lipoproteins and/or peptidoglycans, such as the paramyxovirus hemagglutinin-neuraminidase (HN) envelope protein [60, 61]. However, in this study, only rSeV-GFP-P25 induced the upregulation of TLR6 compared with the basal expression found in untreated cells. Previously, the HIV protein p24 was shown to stimulate the TLR2/6 complex [62], and this complex is overexpressed in SRLV-infected animals with high proviral loads [63], suggesting that the SRLV recombinant protein P25 can be sensed by TLR2/6. However, most studies have suggested that SRLV is recognized by TLR3, 7, 8 or 9 [21, 63–66]. In this work, more rSeV-GFP-P25 than SeV-GFP induced the upregulation of TLR3 and TLR7, suggesting that P25 RNA could also be sensed by TLR3 and TLR7. Additionally, compared with SeV-GFP, rSeV-GFP-P25 induced greater overexpression of all PRRs at 72 hpt, suggesting that the induction of innate responses in ovine cells after SeV infection is likely enhanced by P25 protein expression [48]. Nevertheless, direct comparison between SeV-GFP and rSeV-GFP-P25 did not reveal statistically significant differences.

Upregulation of ovine APOBEC3Z1 (A3Z1), OBST2 and SAMHD1 antiviral factors was observed in OSF transduced with both SeV-based vectors. The activation of these ISGs has been associated with SRLV restriction in vitro [17–19, 48, 62]. The A3Z1 isoform contains one cytidine deaminase motif that is able to induce detrimental G-to-A mutations in viral DNA before integration [68]. Increased A3Z1 expression has been related to restricted SRLV replication in wild ruminants as well as in resistant goats [17, 67]. Interestingly, A3Z1 is resistant to *Vif*-mediated degradation, in contrast to other isoforms, and is highly expressed in restrictive cells for SRLV replication, such as monocytes or M1 macrophages [17, 20]. The expression of A3Z1 quickly increased after SeV-GFP transduction and decreased below the limit marked by untransduced OSF at 72 h. In the case of rSeV-GFP-P25, although not statistically significant, A3Z1 expression was delayed but maintained, suggesting a role for P25 in the upregulation of this restriction factor. Similar expression kinetics after SeV-based vector transduction were observed in the case of SAMHD1, which can reduce the dUTP available for lentivirus replication [69]. However, studies on the SRLV are lacking. OBST2/Tetherin was the most upregulated antiviral factor compared with untransduced cells, with significant differences between SeV-GFP and rSeV-GFP-P25. OBST2/Tetherin has emerged as an important molecule for host cell defense by inhibiting the release and spread of lentiviruses and other enveloped viruses [70]. Taken together, these results highlight the inhibitory roles of ISGs individually or in conjunction to restrict SRLV in vitro. Further studies are necessary to understand which type of IFN or cytokine is involved in the SeV-based vector-induced innate immune response.

To characterize SRLV restriction in OSF upon transduction with the two SeV-based vectors, we quantified the virus DNA load at different times post transduction and post-infection. SRLV restriction gradually increased after transduction with SeV-GFP or rSeV-GFP-P25 at 24, 48, 72 and 96 hpt. Therefore, these results suggest that the protective capacity of the innate immune response induced by these two SeV-based vectors has a longer effect than does transgene expression. Additionally, this restrictive capacity was maintained at 24, 48 and 72 hpi with SRLV, whereas untransduced cells presented a progressive increase in viral DNA. Moreover, OSF transduced three times with SeV-GFP or rSeV-GFP-P25 presented significantly lower viral DNA loads than OSF transduced one or two times did, demonstrating a cumulative effect, likely referred to as the memory of innate immunity exerted by epigenetic modifications [71, 72]. Interestingly, no significant differences were observed between SeV-GFP and rSeV-GFP-P25 in terms of in vitro SRLV restriction. Nevertheless, differences may appear in vivo when adaptive immunity occurs. Given the high genetic diversity among SRLV genotypes, future research will assess the antiviral response of rSeV-GFP-P25-transduced cells against SRLVs from genotype B.

To evaluate the potential antiviral response induced after transduction of OSF with SeV-GFP or rSeV-GFP-P25, the supernatants were collected and added to fresh OSF, which was subsequently infected with SRLV. The viral DNA load was reduced in OSF incubated with these supernatants at all hpt, with significant differences being more evident at 48 hpt. There were no statistically significant differences when comparing empty SeV-GFP with rSeV-GFP-P25; however, in contrast with rSeV-GFP-P25, SeV-GFP was unable to induce IFN-β upregulation in OSF. Therefore, other type-I IFNs (IFN-α), type-II IFNs (IFN-γ), IFN-λ and inflammatory cytokines may play a role in inducing antiviral responses against SRLV [16, 73]. These results suggest that the antiviral response induced by SeV-GFP and rSeV-GFP-P25 is not exclusively based on the IFN-β-mediated response.

The present study revealed efficient and transient expression of the recombinant SRLV P25 protein in OSF transduced with rSeV-GFP-P25. Moreover, marked P25 protein expression in the ciliated epithelium of the nasal mucosa was observed in the lamb inoculated with rSeV-GFP-P25. The macrophages/DCs of the submucosa also showed positive cytoplasmic immunolabelling. The induction of strong transgene expression during viral vector-based immunization is necessary to trigger a specific immune response [74]. Therefore, P25 expression in submucosal antigen-presenting cells of the nasal cavity could indicate the eventual activation of adaptive immunity. In this study, lambs inoculated with SeV-GFP and rSeV-GFP-P25 presented a marked local reaction at the inoculation point characterized by hyperplasia of ciliated epithelial cells, moderate infiltration of lymphocytes into the nasal submucosa and marked reactive lymphoid hyperplasia in the NALT at 48 h post-inoculation, suggesting early and efficient SeV transduction. However, pathological findings were absent in the lower respiratory tract. Although transgene expression may vary between animals, these preliminary results demonstrate the expression of recombinant protein in sheep inoculated with a recombinant SeV vector, as previously observed [47], suggesting that rSeV-GFP-P25 could induce an adaptative immune response against strain Ev1 of SRLV.

In summary, SeV-GFP and rSeV-GFP-P25 were successfully generated using the SeV reverse genetics system. rSeV-GFP-P25 induced efficient transgene expression in vitro and in vivo, robust antiviral innate immune responses and partial restriction of SRLV infection in ovine skin fibroblasts. This study reinforces the important role of the innate immune response in the control of SRLV infection. Future research will clarify the level of protection induced by rSeV-GFP-P25 in sheep after SRLV experimental challenge.

## Supplementary Information

**Additional file 1. Primer and probe sequences used in the study.**

## Data Availability

All the data generated or analysed during this study are included in this published article [and its supplementary information files].
