# Supplementary material for: Characterization of a recombinant Sendai virus vector encoding the small ruminant lentivirus gag-P25: antiviral properties in vitro and transgene expression in sheep
Source: Vet Res. 2025 Mar 7;56:51. doi: 10.1186/s13567-025-01475-2 (PMC11889777; doi:10.1186/s13567-025-01475-2)
Supplement: Supplementary file 1 — Additional file 1. Primer and probe sequences used in the study. [file 13567_2025_1475_MOESM1_ESM.docx]

**SUPPLEMENTARY MATERIAL**

**Characterization of a recombinant Sendai virus vector encoding small ruminant lentiviruses *gag*-P25: antiviral properties *in vitro* and transgene expression in sheep**

Álex Gómez^1,2^, Idoia Glaria^3^, Irati Moncayola^3^, Irache Echeverría^4^, Javier Arrizabalaga^3^, Ana Rodríguez-Largo^1^, Ignacio de Blas^1,2^, Delia Lacasta^1,2^, Estela Pérez^1,2^, Marta Pérez^2,5^, Alicia De Diego^6^, Ricardo De-Miguel^7^, Benhur Lee^8^, Lluís Luján^1,2^, Ramsés Reina^3^*****

^1^Departamento de Patología Animal, Universidad de Zaragoza, Zaragoza, Spain.

^2^Instituto Agroalimentario de Aragón-IA2, Universidad de Zaragoza, Zaragoza, Spain.

^3^Instituto de Agrobiotecnología (CSIC-Gobierno de Navarra), Mutilva, Navarra, Spain.

^4^Departamento de Agronomía, Biotecnología y Alimentación, Universidad Pública de Navarra, Pamplona, Spain.

^5^Departamento de Anatomía, Embriología y Genética Animal, Universidad de Zaragoza, Zaragoza, Spain.

^6^Instituto Aragonés de Ciencias de la Salud (IACS), Centro de Investigación Biomédica de Aragón (CIBA), Zaragoza, Spain.

^7^AnaPath Services GmbH, Liestal, Switzerland

^8^Department of Microbiology, Icahn School of Medicine at Mount Sinai, New York, New York, USA

**Supplementary Table S1**. Primer and probe sequences used in the study.

| Name | Primer sequence (5´–3´) | Descriptions | Source |
| --- | --- | --- | --- |
| Gag-Pol | Fw 1: TGGTGARKCATAGMTAGAGACATGG | Used in SRLV diagnostic |  |
|  | Fw 2: CAAACWGTRGCAATGCAGCATGG |  |  |
|  | Rv 1: CATAGGRGGHGCGGACGGCASCA |  | [75] |
|  | Rv 2: GCGGACGGCASCACACG |  |  |
| Craft-Oslo | Fw: TGACAGAAGGAAATTGTYTRTGG |  | [76] |
|  | Rv: GGCATCATGGCTAATACTTCTAA |  |  |
| EV1 | Fw: CTCCTTGCAGGCCACAATG | Used in evaluation of proviral load *in vitro* assays |  |
|  | Rv: GCTGCTTGCACTGTCTCGG |  | [36] |
| EV1 probe | 6-FAM-TGCCTTATGTGTAGTCAGC-TAMRA |  |  |
| P25 NotI IF | Fw: GTGGTGACAGCGGCCGCGTCTTCCAGCAATTGCAAACTGTG | Used to clone EV1 P25 into rSeV-GFP | This study |
|  | Rv: TCCGGATCCGCGGCCGCAAAACCCTTCTGATCCTACATCTCTACATG |  |  |
| EV1 P25 | Fw: CTTGTGTCCGAGGATTTTGA | Used in transgene expression *in vitro* and *in vivo* | [55] |
|  | Rv: CCCACCTTTCTGCTTCTTCATT |  | This study |
| TLR1 | Fw: CCCACAGGAAAGAAATTCCA | Used in gene expression |  |
|  | Rv: GGAGGATCGTGATGAAGGAA |  |  |
| TLR2 | Fw: CCGAAAGCACAAAGATGGTT |  |  |
|  | Rv: ACGACGCCTTTGTGTCCTAC |  | [77] |
| TLR3 | Fw: GAGGCAGGTGTCCTTGAACT |  |  |
|  | Rv: GCTGAATTTCTGGACCCAAG |  |  |
| TLR4 | Fw: TGGATTTATCCAGATGCGAAA |  | This study |
|  | Rv: GGCCACCAGCTTCTGTAAAC |  |  |
| TLR5 | Fw: CATCAGATGGAACTGGGACA |  |  |
|  | Rv: AAAACCACATCGCCAACATC |  |  |
| TLR6 | Fw: GTTTTCCCAGTCACGAC |  |  |
|  | Rv: CAAAGCAGGGAACAATCCAT |  |  |
| TLR7 | Fw: GCTGGAGAGATGCCTGCTAT |  |  |
|  | Rv: ACTCCTTGGGGCTAGATGGT |  | [77] |
| TLR8 | Fw: GGTCCCAATCCCTTTCCTCTA |  |  |
|  | Rv: TCCACATCCCAGACTTTCTACGA |  |  |
| TLR9 | Fw: CACCTCCGTGAGGTTGTTGT |  |  |
|  | Rv: CTCGTATCCCTGTCGCTGAG |  |  |
| TLR10 | Fw: TCTGCCTGGGTGAAGTATGA |  |  |
|  | Rv: AATGGCACCATTCAGTCTGG |  |  |
| RIG-I | Fw: GCTGACGGCCTCAGTTGGT |  | [48] |
|  | Rv: TCGAGAGAAGCACACAGTCTGC |  |  |
| MyD88 | Fw: GCATCGAGGAGGACTGCCA |  | This study |
|  | Rv: CCGAGGGATGCTGCTGTCT |  |  |
| IFN-β | Fw: GATGCCGTATTGGTCATGTA |  |  |
|  | Rv: CATCTGCCCATAGAGTTCCT |  |  |
| A3Z1 | Fw: TCCGTTCTTGGAATCTGGAC |  | [20] |
|  | Rv: GTATAGATGCGGGAGGCAAA |  |  |
| OBST2 | Fw: CGTGGACGGCCTCCAAG |  | [48] |
|  | Rv: TGGCAGCTTCGGCTTCC |  |  |
| SAMHD1 | Fw: GAGAACGAAGCTGCTTAATTGTATCC |  |  |
|  | Rv: GAGGTGTGTCGATGATTCGGA |  |  |
| β-actin | Fw: CTCACGGAGCGTGGCTACA | Used in transgene and gene expression | [78] |
|  | Rv: TACGTGGGAAGCGCCTCGCT |  |  |

**References**

75. Grego E, Bertolotti L, Quasso A, Profiti M, Lacerenza D, Muz D, Rosati S (2007) Genetic characterization of small ruminant lentivirus in Italian mixed flocks: evidence for a novel genotype circulating in a local goat population. J Gen Virol 88:3423-3427

76. Rimstad E, East NE, Torten M, Higgins J, DeRock E, Pedersen NC (1993) Delayed seroconversion following naturally acquired caprine arthritis-encephalitis virus infection in goats. Am J Vet Res 54:1858-62

77. Menzies M, Ingham A (2006) Identification and expression of Toll-like receptors 1-10 in selected bovine and ovine tissues. Vet Immunol Immunopathol 109:23-30

78. Reina R, Glaria I, Benavides J, de Andrés X, Crespo H, Solano C, Pérez V, Luján L, Pérez MM, Pérez de la Lastra JM, Rosati S, Blacklaws B, Harkiss G, de Andrés D, Amorena B (2007) Association of CD80 and CD86 expression levels with disease status of Visna/Maedi virus infected sheep. Viral Immunol 20:609-22
